# Metagenome-Assembled Genomes of Pig Fecal Samples in Nine European Countries: Insights into Antibiotic Resistance Genes and Viruses

**DOI:** 10.3390/microorganisms12122409

**Published:** 2024-11-24

**Authors:** Boxuan Yang, Jianbo Yang, Routing Chen, Jianmin Chai, Xiaoyuan Wei, Jiangchao Zhao, Yunxiang Zhao, Feilong Deng, Ying Li

**Affiliations:** 1 Guangdong Provincial Key Laboratory of Animal Molecular Design and Precise Breeding, Foshan University, Foshan 528225, China; boxuanyang04@163.com (B.Y.); jianbo952@gmail.com (J.Y.); crtxxx@139.com (R.C.); jchai@uark.edu (J.C.); 2 School of Animal Science and Technology, Foshan University, Foshan 528225, China; 3 Department of Animal Science, Division of Agriculture, University of Arkansas, Fayetteville, AR 72701, USA; xw010@uark.edu (X.W.); jzhao77@uark.edu (J.Z.); 4 College of Animal Science & Technology, Guangxi University, Nanning 530004, China; yunxiangzhao@126.com

**Keywords:** metagenome-assembled genomes, antibiotic resistance genes, viruses, pig gut

## Abstract

Gut microbiota plays a crucial role in the health and productivity of pigs. However, the spread of antibiotic resistance genes (ARGs) and viruses within the pig intestinal microbiota poses significant threats to animal and public health. This study utilized 181 pig samples from nine European countries and employed metagenomic assembly methods to investigate the dynamics and distribution of ARGs and viruses within the pig intestinal microbiota, aiming to observing their associations with potential bacterial hosts. We identified 4605 metagenome-assembled genomes (MAGs), corresponding to 19 bacterial phyla, 97 families, 309 genera, and a total of 449 species. Additionally, 44 MAGs were classified as archaea. Analysis of ARGs revealed 276 ARG types across 21 ARG classes, with Glycopeptide being the most abundant ARG class, followed by the class of Multidrug. *Treponema D sp016293915* was identified as a primary potential bacterial host for Glycopeptide. Aligning nucleotide sequences with a viral database, we identified 1044 viruses. Among the viral genome families, Peduoviridae and Intestiviridae were the most prevalent, with *CAG-914 sp000437895* being the most common potential host species for both. These findings highlight the importance of MAGs in enhancing our understanding of the gut microbiome, revealing microbial diversity, antibiotic resistance, and virus–bacteria interactions. The data analysis for the article was based on the public dataset PRJEB22062 in the European Nucleotide Archive.

## Simple Summary

The gut microbiota of pigs plays an essential role in their health and growth. Observing the connections between antibiotic resistance genes, viruses, and their potential microbial hosts helps in comprehensively understanding the pig gut microbiota. This study aims to use metagenomic assembly and binning techniques to understand the distribution of antibiotic resistance genes and viruses in the pig gut microbiota and to identify possible microbial hosts.

## 1. Introduction

The formation of antimicrobial resistance (AMR) is influenced by multiple factors, with antibiotic usage being the primary one, and it is often conferred to bacteria by ARGs. The presence of resistance genes can lead to reduced effectiveness of antibiotic treatments, impacting the health and welfare of pigs [1,2,3]. In recent years, it has been widely recognized that livestock are an important source of ARGs. This is highlighted in summary reports from the European Union, where particular emphasis is placed on multidrug resistance, complete sensitivity and combined resistance patterns to important antimicrobials, and recommendations from the World Health Organization for Animal Health on monitoring the quantities of antimicrobials used in food animals [4,5]. Significant progress has been made in studying ARGs and tracking their primary transmission pathways. For example, ARGs can be transmitted through pathogens carried in food (such as meat) [6], and as pigs are one of the world’s primary food-producing animals, the ARGs in their gut can be transmitted to humans through the food chain, leading to antibiotic resistance in humans [7].

Moreover, most studies have focused on known single or multiple ARGs of interest [8], overlooking potentially significant but less characterized ARGs. In previous research, regression curves were used to predict ARGs by considering the correlation between multiple variables and establishing statistical models [9]. Therefore, this study utilized high-throughput sequencing of the gut microbiome to construct high-quality metagenomes from 181 pig fecal samples from nine European countries. The aim is to understand the distribution of ARGs in the pig gut microbiota and to identify microbial hosts and their corresponding ARGs. This is crucial for the prevention and control of the increase in antibiotic resistance and the spread of ARGs.

Pig feces is commonly used as fertilizer but may contain residues of antibiotics, ARGs, or viruses, potentially leading to the spread of resistance genes and viruses in the environment. Intestinal viral infections in pigs are prevalent in the global pig farming industry, and the numerous viruses present in the pig intestine can significantly impact the structure and function of the intestinal microbiota. To address this, understanding the intestinal viruses in pigs is significant for improving pig health and production efficiency [10,11,12]. However, the application of metagenomics to the study of intestinal viruses in pigs is not yet comprehensive. Therefore, we utilized metagenomic data from nine European countries to analyze microbial viruses in the intestines of pigs and linked these viral genomes with their potential bacterial hosts. Previously, relatively few studies have used such a large number of fecal samples and MAGs to investigate the relationship between viruses and their bacterial hosts in pigs. This study expands our understanding of the types and functions of viruses and reveals how they impact host health, disease progression, and resistance mechanisms.

Given this context, this study aims to use metagenomic assembly methods to study the distribution of ARGs and viruses in the pig intestinal microbiota, observing their connections with potential bacterial hosts.

## 2. Materials and Methods

### 2.1. Data Collection

To observe the relationship among ARGs, viruses, and potential bacterial hosts, we reanalyzed the metagenomic sequencing dataset of pig fecal samples collected from nine European countries (Belgium (BE), Bulgaria (BG), Denmark (DK), France (FR), Germany (DE), Italy (IT), Netherlands (NL), Poland (PL), and Spain (ES)) that were deposited in the European Nucleotide Archive under the project accession number PRJEB22062 [13]. The following is a brief description of the samples. A total number of 20 traditional integrated farrow-to-finish pig farms were collected between May 2014 and December 2015. These farms are far from livestock trade and are randomly distributed regionally. These pig farms had at least 150 sows and 600 fattening pigs and employed batch production to ensure that the sampled animals came from the same birth cohort. In each pig farm, 25 fresh fecal samples were randomly collected and combined as one sample for the representativeness of the samples. After processing, composite fecal samples from 181 pig feces were obtained [13]. For detailed information on the pig sampling procedure, sample handling and storage, pooling of samples, DNA extraction, and sequencing, readers are directed to Munk et al. [13].

### 2.2. MAG Collection Construction

The pig reference genome (Sus scrofa genome assembly Sscrofa11.1, accession number: GCF_000003025.6) was downloaded from NCBI database. To reconstruct microbial genomes from raw data, we preprocessed these 181 raw data using the KneadData pipeline v0.7.2 [14]. for removal of host contamination [7]. We assembled contigs utilizing the SPAdes v3.15.5 software [15], reducing contamination by minimizing sample cross-talk using bowtie2 v2.5.1 [16]. Binning was then conducted with MetaBAT2 v2.12.1 [17], and quality assessment was performed using CheckM v1.2.2 [18]; it was decided to retain MAGs with ≥50% completeness and ≤10% contamination. Redundant bins were removed using the dRep v3.4.5 software with the parameter ‘-sa 0.99’ [19]. The MAGs were taxonomically annotated using GTDB-Tk v2.2.6 with GTDB release 207_v2 [20]. Using the GTDB parameter ‘gtdbtk infer’, the resulting files were visualized on the iTOL website to produce a phylogenetic tree [21].

### 2.3. ARG Analysis

We constructed complete gene sets from all sample bins using Prodigal v2.6.3 [22], followed by dereplication with CD-HIT v4.8.1 [23]. The dereplicated results were indexed with Salmon [24], and ARG identification was completed using the DeepARG v2 software [25]. Finally, quantification of all genomes was performed with Salmon to calculate the abundance of ARG reads in different samples, assessing the distribution of the same ARG type across similar or identical species among different countries [14].

In the results obtained from DeepARG, we mapped ARGs to MAGs through contig alignment to identify the species associated with the ARGs. To ensure the accuracy of the analysis, we filtered MAGs with completeness > 98% and contamination < 2%. We selected five bacterial species and downloaded their genomes from NCBI (with human, pig, and wild animal hosts, primarily from European locations; genome information is listed in Supplementary Table S1). After Prodigal prediction and DeepARG annotation of these genomes, we clustered ARGs from different hosts using CD-HIT at a 1 similarity threshold.

### 2.4. Virus Analysis

To predict potential bacterial hosts for viruses, we initially clustered the predicted genes to create a nonredundant gene collection using the CD-HIT software with a 95% similarity threshold. Subsequently, we aligned the nonredundant genes to viral gene sequences from the PVD database [26] utilizing BLAST 2.14.0+ [27]. Sequences were retained if they exhibited a score of ≥85% and a length of ≥5 kb. This process ultimately identified 1045 viral genomes [28].

## 3. Results

### 3.1. Metagenome-Assembled Genomes

**Figure 1:**
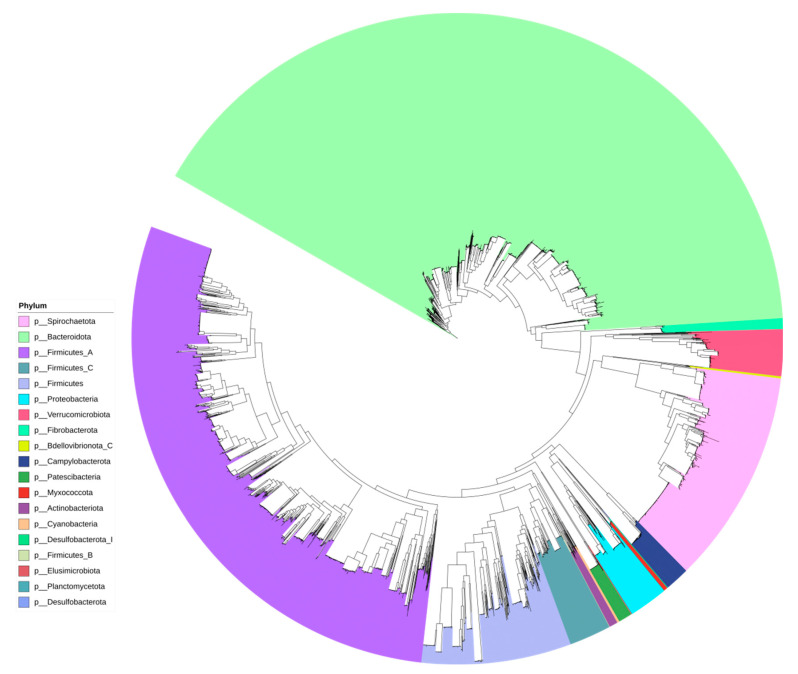
Phylogenetic tree of MAGs of pigs from nine European countries. Phylogenetic tree describing the relationships among MAGs in pig fecal samples from nine European countries, with each color corresponding to a different bacterial phylum.

We obtained 9069 MAGs that satisfied quality thresholds (completeness > 50%, contamination < 10%) (Supplementary Table S2). For the identification of representative MAGs, we utilized de novo clustering with a 99% similarity threshold. After 99% clustering analysis, we obtained 4605 nonredundant MAGs. From the 4605 nonredundant MAGs, we identified 19 bacterial phyla (Figure 1).

Within the classification of these MAGs, the phylum Bacteroidota dominated, comprising 1908 MAGs (or 42%), followed by *Firmicutes A* with 1350 MAGs (or 30%), *Spirochaetota* with 500 MAGs (or 11%), and *Firmicutes* with 348 MAGs (or 8%). At the genus level, the GTDB encompassed 309 bacterial genera, corresponding to these 4561 nonredundant MAGs, with 55 MAGs not having a corresponding genus in the GTDB database. The most abundant genus was *Cryptobacteroides* (490 MAGs), followed by *Treponema D* (400 MAGs), and *Prevotella* (398 MAGs). A total of 449 species were identified, with the most common being *Treponema D porcinum* (146 MAGs), followed by *Cryptobacteroides sp004552115* (100 MAGs), *Alloprevotella sp004552865* (93 MAGs), and *CAG-177 sp003514385* (84 MAGs). The distribution of species varied by country, but aside from BG, *Treponema D porcinum* was widely found across all countries (Figure S1).

Furthermore, through genomic classification databases, 44 MAGs were categorized as archaea, divided into two unique phyla: *Methanobacteriota* and *Thermoplasmatota*. The most abundant species within these archaea were *Methanomethylophilus alvus* and *MX-02 sp006954405*, each represented by 14 MAGs. This classification enriches our understanding of the microbial and archaeal diversity within the pig gut across different European countries, highlighting the complexity and dynamic nature of these microbial communities.

### 3.2. Antibiotic Resistance Gene Host Prediction

We investigated the host diversity of ARG classes and ARG types in pig fecal samples collected from nine European countries. To enhance the accuracy in identifying the potential bacterial hosts of ARGs, we systematically analyzed these MAGs, employing the DeepARG (v2) software for ARG identification. From the nonredundant MAGs, we identified 7125 nonredundant ARGs at a clustering similarity threshold of 99%, including 276 ARG types and 21 ARG classes. Notably, 542 ARGs were unclassified within their ARG class. We visualized the relationships between ARG classes and species across different countries using heatmaps to examine the overall structure and composition of the ARG classes and their potential bacterial hosts.

**Figure 2:**
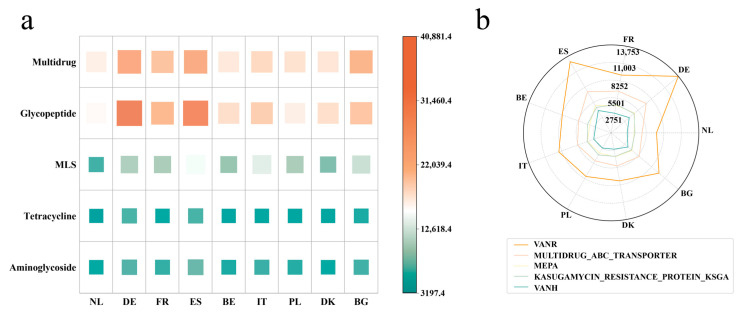
Analysis of resistance genes in MAGs of pigs from nine European countries. (**a**) The TPM of major ARG classes across nine countries. (**b**) The TPM of major ARG types across nine countries. The axes from top to bottom are 13,753; 11,003; 8252; 5501; and 2751.

The relative abundance of ARG types and ARG classes within the pig gut microbiome indicated that in all nine countries, Glycopeptide was the ARG class with the highest abundance, followed by Multidrug and MLS (Figure 2a). Among the ARG types, VANR (ARG type) consistently exhibited significant abundance across all nine countries, followed by MULTIDRUG ABC TRANSPORTER (Figure 2b).

**Figure 3:**
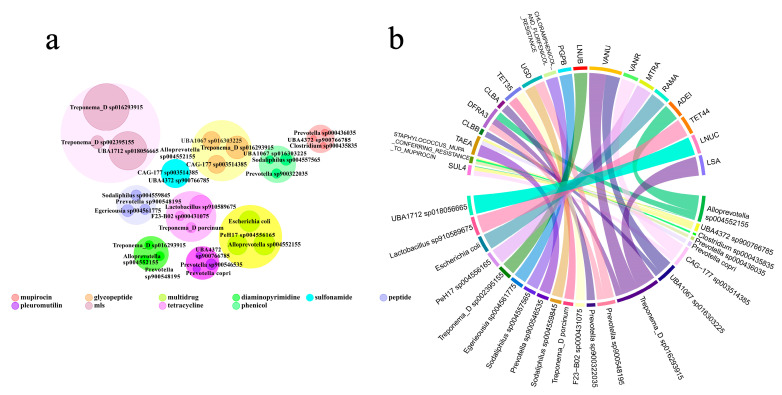
Analysis of ARGs and their potential bacterial hosts. (**a**) The bubble chart shows the relationships between ARG classes and their potential bacterial hosts. The sizes of the bubbles correspond to abundance, with larger bubbles representing ARG classes. Within each large bubble, three smaller bubbles represent the three most abundant potential bacterial hosts within that ARG class. (**b**) Chord diagram from potential bacterial hosts to ARG types. The thickness of the chords corresponds to abundance.

Within the ARG classes, *Treponema D sp016293915* was a major potential bacterial host for the Glycopeptides; the abundance of *Treponema D sp016293915* was 3709.605969, TPM, followed by *CAG-177 sp003514385* (2144.624871, TPM) and *UBA1067 sp016303225* (2111.202327, TPM). *Alloprevotella sp004552155* (3193.277269, TPM) emerged as the main potential bacterial host for Multidrug, followed by *Escherichia coli* (3043.721563, TPM) and *PeH17 sp004556165* (2656.386467, TPM), while *Treponema D sp016293915* (19,286.38244, TPM) was identified as the principal potential bacterial host for MLS, followed by *UBA1712 sp018056665* (12,379.1974 TPM) and *Treponema D sp002395155* (976.931944, TPM) (Figure 3a). We identified *Corynebacterium sp012838985* and *Latilactobacillus curvatus* as potential bacterial hosts for tetracycline and *Corynebacterium sp012838715* and *Lacticaseibacillus sharpeae* as potential bacterial hosts for MLS. Both belong to the same bacterial genus. The bacterial genus *Treponema D*, which had a high abundance, carried a larger number of ARG classes (Figure S2).

Among these potential bacterial hosts, we observed their main ARG types. The primary ARG type for *Treponema D sp016293915* was VANU. The main ARG type for *Alloprevotella sp004552155* was ADEI, and the following major ARG type for *Treponema D sp016293915* was LSA. These potential bacterial hosts correspond to Glycopeptides, Multidrug, and MLS (Figure 3b).

**Figure 4:**
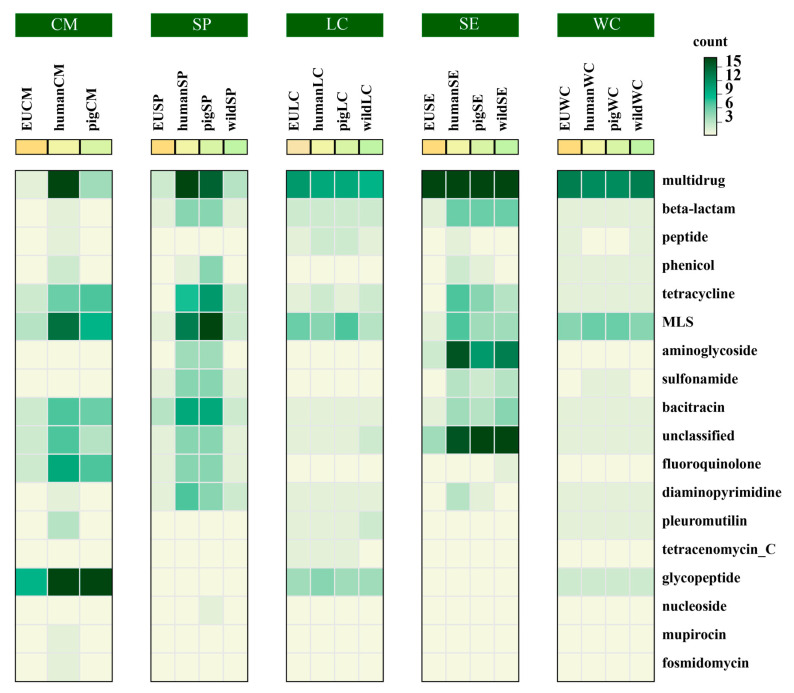
Heatmap of ARGs clustering in different hosts of five species. CM, SP, LC, SE, and WC correspond to *Catenibacterium mitsuokai*, *Streptococcus pasteurianus*, *Latilactobacillus curvatus*, *Salmonella enterica*, and *Weissella cibaria*, respectively. Each species is divided into four groups: EU (the MAGs in this article), human, pig, and wild (wild animals). For *Catenibacterium mitsuokai*, no data for wild animals were downloaded. The y-axis represents the ARG class. The “count” indicates the number of ARGs.

From the DeepARG annotation results, we mapped ARGs back to their corresponding MAGs based on contig alignments. In total, we identified 25 MAGs with completeness > 98% and contamination < 2%. We selected *Weissella cibaria*, *Streptococcus pasteurianus*, *Salmonella enterica*, *Latilactobacillus curvatus*, and *Catenibacterium mitsuokai* as study subjects. In order to study the relationship between ARGs in different hosts, we downloaded the genomes of these species from NCBI, with hosts including pigs, humans, and different wild animals. After gene prediction by Prodigal, we annotated these genomes with DeepARG. ARGs from different hosts were merged and clustered by CD-HIT. We set the CD-HIT similarity threshold to 1, which means that only ARGs that were completely identical across different hosts were clustered together. Wild animals, due to their limited exposure to antibiotic environments used by humans, typically carry fewer ARGs. Therefore, the selection of genomes from wild animals served as a reference in our study. We clustered the ARGs from the five species and found that ARGs within the same cluster were widely present in different hosts across the five species (Figure 4). For example, the Multidrug ARG class demonstrated broad adaptability across different hosts. In contrast, the Glycopeptide ARG class clustered specifically within *Catenibacterium mitsuokai*, indicating that this species may carry more Glycopeptide, showing specificity to this ARG class. Additionally, although ARGs were clustered within the same cluster, ARGs from different species tended to aggregate in different hosts, suggesting that ARG expression may be influenced by the host environment. For instance, in *Streptococcus pasteurianus*, ARGs were more prevalent in humans and pigs than in wild animals, indicating a higher accumulation of ARGs in humans and pigs (Figure 4). This suggests that *Streptococcus pasteurianus* is more likely to carry ARGs and experience ARG transfer in humans and pigs. This observation highlights that both humans and pigs face similar antibiotic selection pressures.

### 3.3. Viral Host Prediction

**Figure 5:**
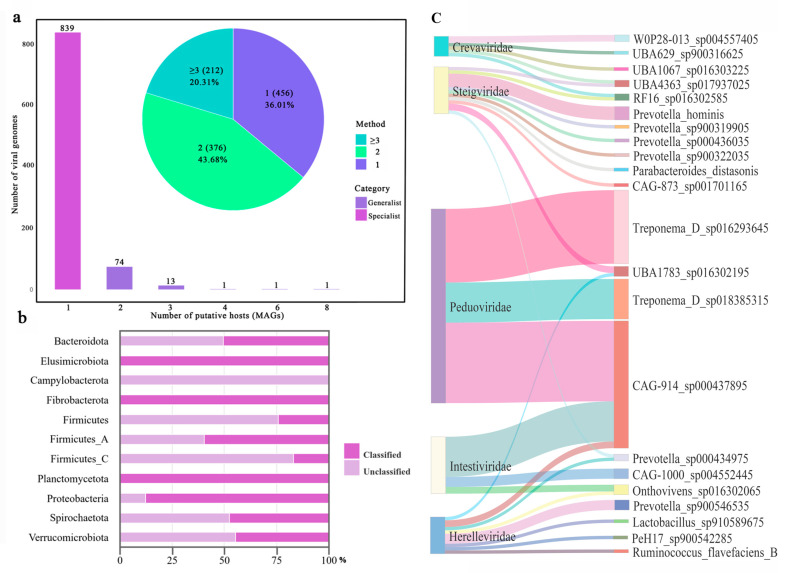
Analysis of viral genomes in MAGs of pigs from nine European countries. (**a**) Bar chart. Viral genomes with only one host were categorized as the Specialist group, and those with two or more hosts were categorized as the Generalist group. The x-axis represents the number of hosts, and the y-axis represents the number of viral genome types. The pie chart shows the methods, including CAT, Demovir, Kaiju, Phagcn, BLASTn, and GeNomad. (**b**) A percentage stacked bar chart shows the proportion of classified and unclassified viral genome families at the phylum level. (**c**) Sankey diagram. The first axis represents the viral genome family. The second axis represents the genus level, illustrating the relationship between bacterial genera and families.

Given that most viruses have species-specific hosts, they serve as ideal tools for precisely manipulating bacteria [29]. In this research, we examined viral genomes within all pig fecal samples that exhibited virus–host interactions, focusing on the connections between these viral genomes and their potential bacterial hosts. This exploration into the interplay between viruses and bacteria in the pig gut microbiome sheds light on the intricate ecological relationships that influence both microbial community dynamics and host health. We aligned preprocessed nucleotide sequences with a comprehensive viral reference database (PVD) [26], considering viral genomes with a BLASTn score of ≥85% and length > 5 kb as indicative of virus–host interactions [30]. Overall, 63.99% of virus–host relationships are supported by more than one method (Figure 5a). A total of 1044 viral genomes exhibiting virus–host interactions were predicted, with 839 viral genomes predicted to have a single host, categorized into the Specialist group, and 90 viral genomes predicted to interact with two or more hosts, categorized into the Generalist group. Among all MAGs, the phylum with the highest allocation was *Firmicutes* (*n =* 351), followed by *Bacteroidota* (*n* = 291), *Firmicutes A* (*n =* 208), *Spirochaetota* (*n =* 137), *Verrucomicrobiota* (*n =* 36), *Proteobacteria* (*n =* 8), *Firmicutes C* (*n =* 6), *Fibrobacterota* (*n =* 3), *Planctomycetota* (*n =* 2), *Elusimicrobiota* (*n =* 1), and *Campylobacterota* (*n =* 1). Within *Firmicutes*, 24.73% of viruses were classified, followed by *Bacteroidota* (49.51%), *Firmicutes A* (47.59%), *Spirochaetota* (62.61%), *Verrucomicrobiota* (87.50%), *Firmicutes C* (16.67%), *Fibrobacterota* (100%), *Planctomycetota* (100%), *Elusimicrobiota* (100%), and *Campylobacterota* (0%) (Figure 5b).

At the genus level, the main genus was *Prevotella* (*n =* 150), followed by *Treponema D* (*n =* 136), *CAG-914* (*n =* 126), and *Lactobacillus* (*n =* 59). These genera play crucial regulatory roles in the structure and function of the pig gut microbiome (Figure 5c).

At the species level, *CAG-914 sp000437895* harbored the most viral genomes, carrying three named viral families and one viral class. It was followed closely by *Onthovivens sp016302065*, which carried three named viral families and one viral class. Subsequently, *UBA1783 sp016302195* carried two named viral families and one viral class. All of them belonged to the class Caudoviricetes.

Among viral genome families, the most numerous was Unclassified (*n =* 594), followed by Peduoviridae (*n =* 371), Intestiviridae (*n =* 20), Herelleviridae (*n =* 15), Steigviridae (*n =* 14), Crevaviridae (*n =* 7), Suoliviridae (*n =* 4), Autographiviridae (*n =* 3), Duneviridae (*n =* 3), etc. The species most frequently allocated within Unclassified was *Onthovivens sp016302065* (*n =* 35); for Peduoviridae, it was *CAG-914 sp000437895* (*n =* 24); and for Intestiviridae, it was *CAG-914 sp000437895* (*n =* 12).

## 4. Discussion

The gut microbiota plays a crucial role in the overall development and metabolic needs of pigs, influencing nutrient digestion, disease resistance, and the production of vitamins and beneficial metabolites, thereby affecting the growth and development of pigs [31,32,33,34,35]. Furthermore, the composition of the gut microbiome undergoes changes during the growth of pigs, with notable differences observed within the intestinal tract. This research is not only vital for animal health but significant for understanding the effects of dietary changes, antibiotic usage, and gut microbiota stress [36]. Many bacterial strains found in the mammalian gut are challenging to culture and isolate because of their unknown growth and nutritional requirements [37]. In this study, we utilized metagenomic assembly to collect and analyze 9069 MAGs, further refining and expanding the reference MAGs with a standalone assembly method that has been proven to produce high-quality genomes [19,38]. We identified 4605 nonredundant MAGs, primarily from the *Bacteroidota*, *Firmicutes A*, and *Spirochaetota* phyla. Metagenomic methods significantly enhance the understanding of the complexity and functionality of the gut microbiota. Combined with previous research, this approach enriches the comprehensiveness of pig gut microbiome research [13].

We adopted MAGs to explore the potential bacterial hosts of ARGs. Metagenomic assembly and binning provide more comprehensive and precise ARG prediction results, especially at the gene level comparison. Assembling longer contigs reduces the likelihood of nonspecific mapping, thereby increasing the accuracy of ARG and virus prediction.

Antimicrobial resistance is increasingly recognized as a severe global health threat [39]. The existence and spread of ARGs among humans and farm animals have been well documented [40]. We explored the relationship between ARGs and their potential bacterial hosts using a MAG assembly approach. This method promotes the investigation into the connection between ARGs and potential bacterial hosts within the pig microbiome. The main ARG classes predicted were Glycopeptides, Multidrug, and MLS, with *Treponema D sp016293915* and *Treponema D sp002395155* showing high abundance in these classes. The *Treponema D* genus was also significantly enriched in other ARG classes. Further research on the *Treponema D* genus is recommended to uncover its drug resistance mechanisms and gene functions.

The prevalence of antibiotics correlates with the usage of antibiotic medications. Interactions between different ARGs, such as those between tetracycline and macrolides, have been identified [41,42,43]. In the tetracycline and MLS (macrolide–lincosamide–streptogramin) classes, certain bacterial hosts were identified within the same genus. For instance, *Corynebacterium sp012838985* and *Corynebacterium sp012838715* were linked to tetracycline and MLS, respectively, and both belong to the Corynebacterium genus. Similarly, Latilactobacillus curvatus and Lacticaseibacillus sharpeae were associated with tetracycline and MLS, respectively, and both belong to the Lacticaseibacillus genus.

The presence of ARGs in the gut microbiota not only raises concerns for animal health but poses significant risks to human health due to the potential transfer of these genes through the food chain and the environment [44]. Additionally, the standardized results of ARGs indicate that Germany (DE) and Spain (ES) had a higher abundance of ARGs. The abundance of ARGs is related to the use of antibiotics [9]. Unfortunately, we lack data on antibiotic use in livestock for each country, but we suspect a link to antibiotic use [45]. Antibiotics are tied to the increase in ARGs. The high levels of ARGs in pig feces indicate a need for stronger policy and enforcement in these countries. These findings remind us that countries need to adopt stricter and more effective measures in the management and use of antibiotics. This also provides a basis for further research on the relationship between antibiotic use and ARGs abundance.

In this study, we adopted an integrative approach to explore the relationship between ARGs and specific species. Through DeepARG annotation, we successfully identified contigs associated with ARGs from metagenomic data and mapped these contigs back to their corresponding MAGs. We downloaded genomes from different hosts from NCBI, setting a wild animal group as a reference. Microbes in livestock and humans are more likely to carry ARGs because of their hosts’ frequent exposure to antibiotics (for disease treatment or growth promotion). In contrast, wild animals, with less exposure to human-used antibiotics, generally carry fewer ARGs. In *Streptococcus pasteurianus*, ARGs clustered within the same cluster showed a higher concentration in humans and pigs than in wild animals, indicating that humans and pigs face similar antibiotic selection pressures. This may facilitate the transfer of ARGs between humans and pigs.

The usage of metagenomics to explore the composition of specific microbial and viral communities has increasingly attracted the attention of researchers. The advancement of high-throughput sequencing technologies has facilitated the discovery of previously unknown microbes, reducing the dependence on traditional culturing methods or specialized separation techniques [46]. To explore which viruses were present in nucleic acid sequences and the relationships between the viruses and their potential bacterial hosts, we aligned nucleotide sequences with viral databases, yielding a total of 1044 viral genomes categorized into four classes and 17 families. Understanding the distribution of viruses and ARGs in gut microbiota is significant for devising strategies to mitigate the spread of resistance genes while maintaining livestock health and productivity.

The study is subject to several limitations: (1) MAGs derived from metagenomic assembly may contain errors, potentially resulting in minor deviations in the research outcomes. Especially, the binning step could take incorrect contigs into a MAG, which would significantly affect the results of this study. However, we removed the higher-contamination-rate MAGs. (2) The investigation of low-abundance bacterial populations presents substantial challenges for researchers, and our study is no exception. The MAG-based method frequently fails to capture low-abundance species and genes, resulting in the omission of critical information. (3) The completeness of ARG and virus databases may impact the accuracy of the research results. In future research, to avoid errors introduced by second-generation sequencing assembly, third-generation sequencing can be used.

## 5. Conclusions

This study employed metagenomics to explore the relationships between ARGs, viruses, and their potential bacterial hosts across metagenomic sequencing data collected from nine European countries. Among 4605 nonredundant MAGs, *Treponema D sp016293915* carried the highest counts of ARGs, while *CAG-914 sp000437895*, *Onthovivens sp016302065*, and *UBA1783 sp016302195* carried the highest counts of viral genomes. MAGs offer more accurate predictions of ARG hosts than previous methods, enhancing our understanding of ARGs in the gut microbiome. This study enhances our understanding of the interactions between ARGs, viruses, and their potential microbial hosts of pigs.

## Data Availability

The code used in this study is publicly available on GitHub at the following link: https://github.com/xuanxuan69/MAG/tree/main (accessed on 18 September 2024). The dataset supporting the findings of this study is available in the figshare repository, https://doi.org/10.6084/m9.figshare.27722280.v1 (accessed on 18 September 2024). The datasets supporting the findings of this study are available in the NCBI database with the accession numbers PRJNA1185935, PRJNA1186098, PRJNA1186113, PRJNA1186118, PRJNA1186124, PRJNA1186133, PRJNA1186134, PRJNA1186142, PRJNA1186148, PRJNA1186149, PRJNA1186151, and PRJNA1186151. The datasets are accessible to all interested researchers for the purpose of reproducing the results or for further analysis.

## Supplementary Materials

The following supporting information can be downloaded at: https://www.mdpi.com/article/10.3390/microorganisms12122409/s1, Figure S1: The counts of different species in nine countries, each color corresponds to a different bacterial species.; Figure S2: Sankey diagram of Treponema D and ARG classes. The thickness of the bands corresponds to the abundance.; Table S1: ncbi_message; Table S2: MAGs_message.
