# Supplementary figures and images for: Metagenome-Assembled Genomes of Pig Fecal Samples in Nine European Countries: Insights into Antibiotic Resistance Genes and Viruses
Source: Microorganisms. 2024 Nov 24;12(12):2409. doi: 10.3390/microorganisms12122409 (PMC11676251; doi:10.3390/microorganisms12122409)

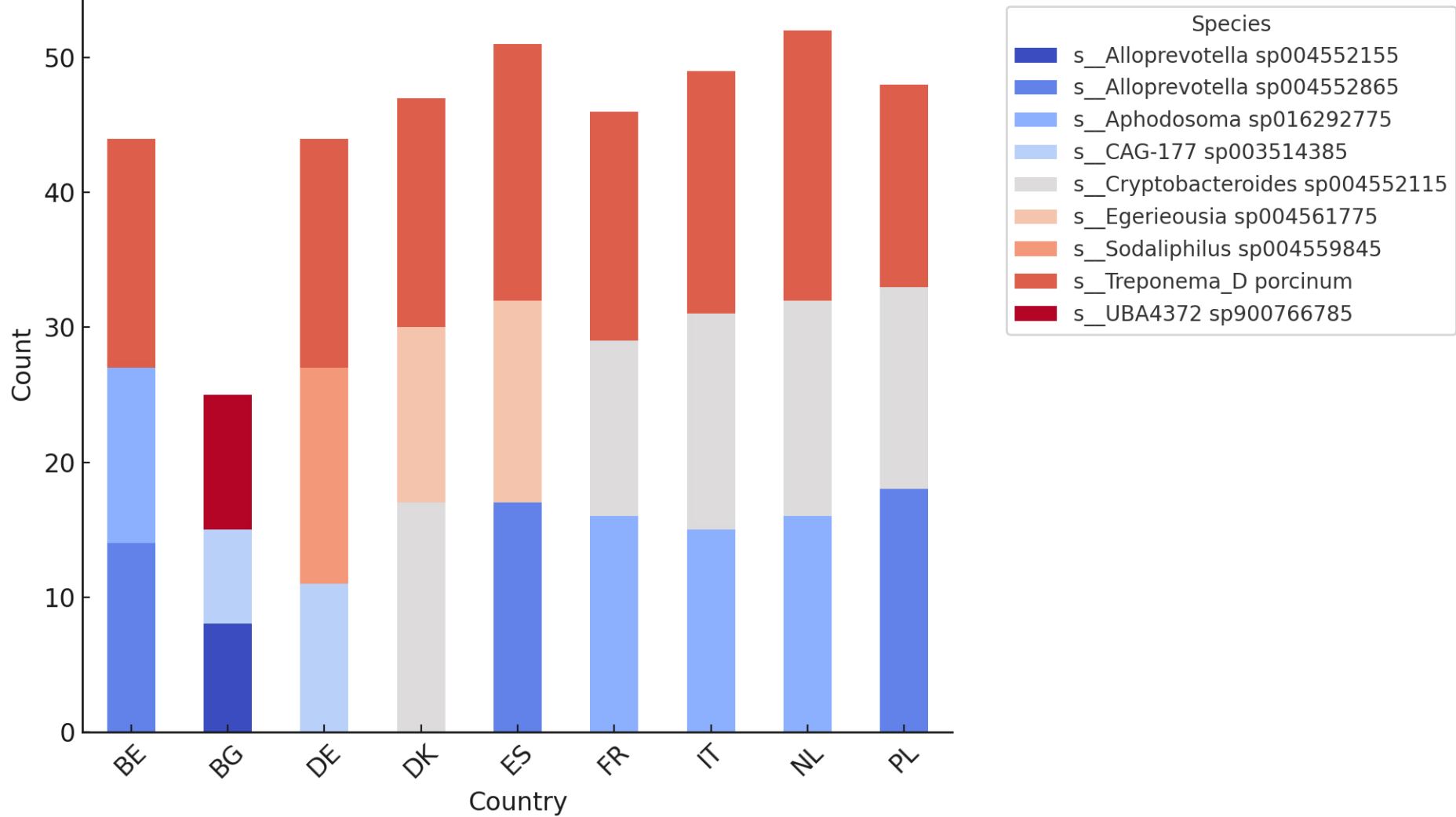

Supplement: Supplementary file 1 [file microorganisms-12-02409-s001.zip › Figure S1 The counts of different species in nine countries.pdf]

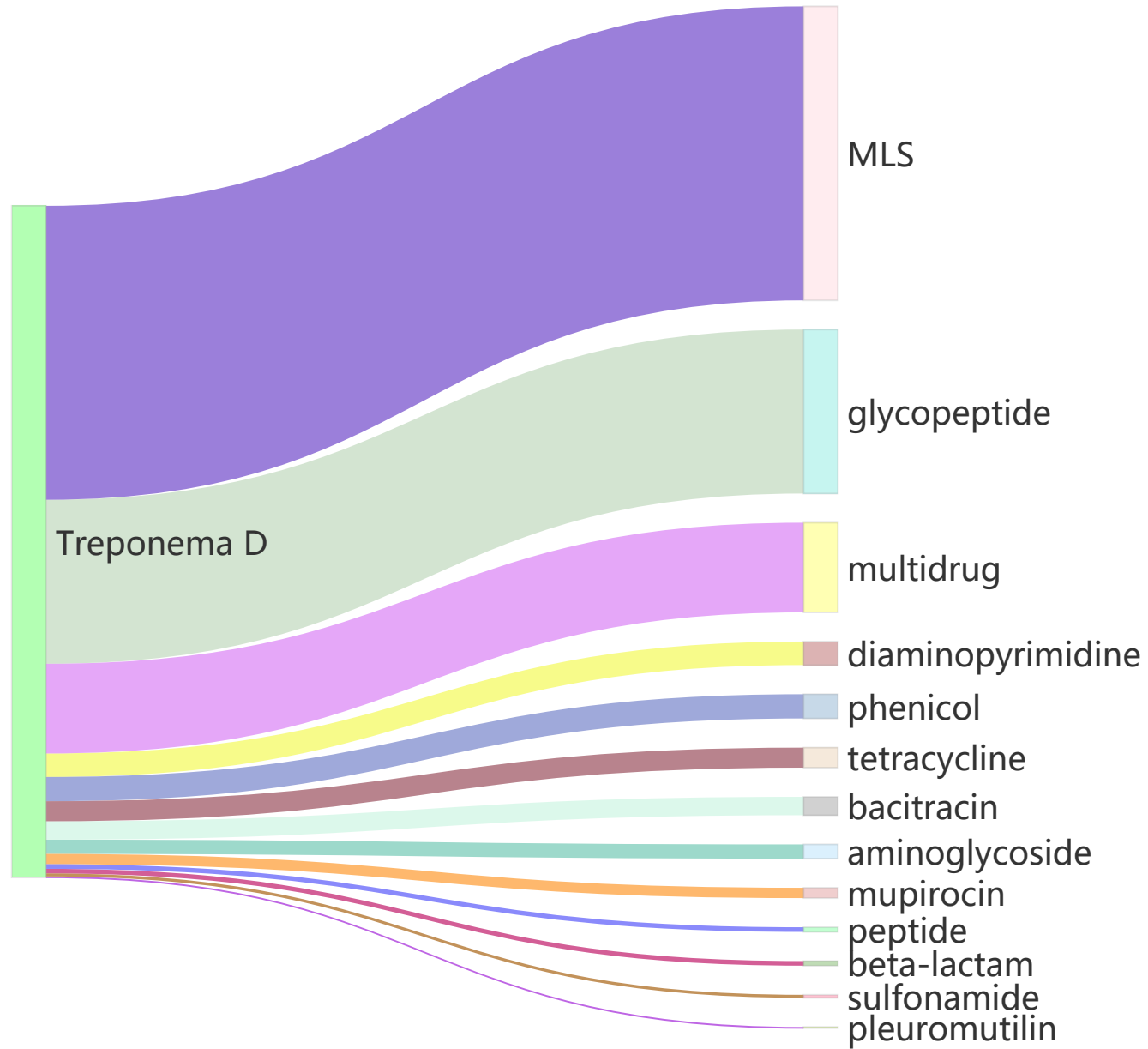

Supplement: Supplementary file 1 [file microorganisms-12-02409-s001.zip › Figure S2 Sankey diagram of Treponema D and ARG classes.pdf]
